# The production of an efficient visible light photocatalyst for CO oxidation through the surface plasmonic effect of Ag nanoparticles on SiO_2_@α-Fe_2_O_3_ nanocomposites

**DOI:** 10.1039/c7ra13260c

**Published:** 2018-04-05

**Authors:** Kasimayan Uma, Shih-Wen Chen, Nadarajan Arjun, Guan-Ting Pan, Thomas C.-K. Yang

**Affiliations:** Centre for Precision Analysis and Research Center, National Taipei University of Technology Taipei Taiwan 106; Department of Chemical Engineering and Biotechnology, National Taipei University of Technology Taipei Taiwan 106 ckyang@mail.ntut.edu

## Abstract

A process for the photo deposition of noble Ag nanoparticles on a core–shell structure of SiO_2_@α-Fe_2_O_3_ nanocomposite spheres was performed to produce a CO photo oxidation catalyst. The structural analyses were carried out for samples produced using different Ag metal nanoparticle weight percentages on SiO_2_@α-Fe_2_O_3_ nanocomposite spheres by X-ray diffraction (XRD), field emission-scanning electron microscopy (FE-SEM), UV-vis spectroscopy, Raman spectroscopy and Fourier transform infrared spectroscopy (FTIR). A computational study was also performed to confirm the existence of the synergic effect of surface plasmon resonance (SPR) for different weight percentages of Ag on the SiO_2_@α-Fe_2_O_3_ nanocomposites. The mechanism for CO oxidation on the catalyst was explored using diffuse reflectance infrared Fourier transform spectroscopy (DRFIT). The CO oxidation results for the Ag (2 wt%)-SiO_2_@α-Fe_2_O_3_ nanocomposite spheres showed 48% higher photocatalytic activity than α-Fe_2_O_3_ and SiO_2_@α-Fe_2_O_3_ at stable temperature.

## Abstract 2

We present a systematic investigation of CO oxidation and surface plasmon resonance on SiO_2_@α-Fe_2_O_3_ nanocomposite spheres with different weight percentages of Ag nanoparticles.

## Graphical abstract

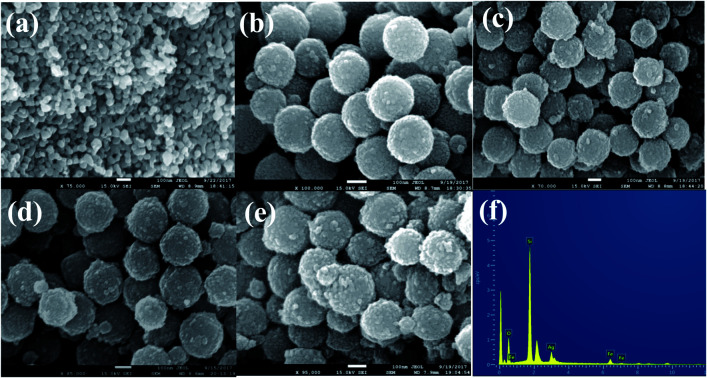

## 1. Introduction

Nowadays interest in the use of photocatalytic processes for the conversion of solar energy to chemical energy has been growing rapidly, being seen as a means to save our environment by reducing the production of greenhouse gases. Light absorption and the utilization of photoinduced electron and hole charge carriers are necessary for solar energy conversion. This conversion process includes plasmonic effects, which are used for applications such as solar water splitting, photo oxidation and degradation of organic pollutants.^1,2^ Carbon monoxide (CO) oxidation is one of the most effective reactions for the remedying of everyday environmental problems and as a consequence has received much scientific interest.^3–7^ For efficient conversion for CO oxidation, a metal oxide is needed, and many conditions need to be satisfied related to the loading of the metal, co-catalysts and surface adsorption.^5,8–11^ It has been found that the Fe and Co based catalysts are more suitable for CO_2_ conversion and hydrocarbon removal.^12^ Metals such as Ag, Au and Cu are the most easily adoptable materials for CO oxidation applications and for effective adsorption on the surface of semiconductor materials. These metals are all important photocatalyst for the oxidation of CO and can be applied for improving organic photo oxidation.^13,14^ Recently, researchers have begun to focus on Ag-based catalysts in particular. This material has attracted intense attention because it is inexpensive and abundant in nature. Different weight percentages of Ag and different sizes of semiconductor nanostructures have been studied to examine the reduction in the recombination rate of photo-generated charges and the performance of different types of photocatalytic activity.^15^ In comparison with Au, Pt and Cu, Ag produces stronger plasmonic effects which enhance CO oxidation along with the advantages of low cost and higher productivity. For example, Ag or Au/TiO_2_ nanorods have been used for CO oxidation and CO_2_ reduction under visible light, which enhances photocatalytic activity due to the surface plasmonic resonance effect (SPR).^16–22^ The Ag based catalysts have attracted more study because of the greater surface area and better catalytic activity which are needed to achieve reasonable conversion rates of CO_2_ from CO.

Recently, the metal doped n-type semiconductor catalysts have played the dominant role in photocatalytic oxidation applications, due to their nontoxicity and enormous variety of applications in industry. Further, the metal oxides, such as TiO_2_, Fe_2_O_3_ and ZnO, which have been extensively studied as stable compounds for the prevention of dye degradation and are relatively cheaper than non-oxide semiconductors, could potentially be adapted for photodegradation and CO oxidation.^23–28^ Among the metal oxides, Fe_2_O_3_ has been shown to have the properties of being a visible light photocatalyst, earth abundant, non-toxic, and photo chemically stable.^29^ This visible light catalyst creates more excited charges and reduces the electron–hole recombination rate which is useful for improving photodegradation of inorganic pollutants and CO oxidation.^30–32^ Although the photocatalytic oxidation of carbon monoxide has been reported in many studies, there have been only a few papers about α-Fe_2_O_3_ core–shell structure.^29,33^ Many types of core–shell materials can be used for the development of visible light response in nanostructures due to SPR effect. Many have used SiO_2_ as a shell material because of its superior chemical and photochemical stability.^34^ Increasingly, Ag modified SiO_2_ core shell nanoparticles have been shown to have upgraded optical properties.^35,36^ The core and thin shells of the nanoparticle structure could be expected to show excellent reactivity and stability for chemical looping and increased adsorption properties. This type of SiO_2_ layer has the advantage of being a template free structure, with a higher adsorption capacity, so has more often been used as a core layer in recent years. Over the past decades, Fe_2_O_3_ has been successfully prepared by various methods to form different structures, such as cubes, rods, needles, wires, tubes, belts, disks, flakes, hollow spheres.^37–41^ Recently the preparation of self-assembled hematite hierarchical structures with promising applications in many areas using a simple template free sol–gel method has been reported.^33,37,40–42^ The SPR intensity and wavelength can be enhanced using hematite nano structure and coupling effect of nano particles such as Ag or Au.^18,43^ However, a clear understanding and evidence of the mechanism of SPR on the photolytic surface of Ag-SiO_2_@α-Fe_2_O_3_ nanocomposites are still needed.

In this work, the objective is to study the efficiency of CO oxidation and formation of hydrocarbons on SiO_2_@α-Fe_2_O_3_ nanocomposite spheres with different weight percentages of Ag nanoparticles. The SPR effects were observed on SiO_2_@α-Fe_2_O_3_ nanocomposites spheres. This phenomenon is proved with the computational method. In addition, the photocatalytic abilities of these products were investigated at various temperatures and almost with constant pressure using DRIFT.

## 2. Experimental method

### 2.1 Materials

The TEOS (tetraethylortho silicate), anhydrous ethanol, ammonia, iron(iii) chloride hexahydrate, urea and AgNO_3_ were purchased form the Aldrich. All the chemicals were analytical grade and were used as received.

### 2.2 Fabrication of Ag-SiO_2_@α-Fe_2_O_3_ nanocomposites spheres

For the preparation of the SiO_2_ nanospheres, we followed the procedure detailed in our previous work.^44^ Briefly, 5 mL of TEOS were mixed with 25 mL anhydrous ethanol and 5 mL water. After 30 min of stirring, 1 mL of ammonia solution was added and stirring extended for another 10 min. Then, 15 mL of anhydrous ethanol were added and the solution stirred continuously for 12 h. Finally, the resulting solid SiO_2_ spheres were washed copiously with ethanol and water using centrifugation. They were then dried in a vacuum oven at 60 °C for 10 h to remove trace amount of organic solvents. Subsequently, 100 mg of SiO_2_ spheres were mixed with 50 mg of iron(iii) chloride hexahydrate and 30 mg of urea then stirred continuously for 8 h at 95 °C. After stirring, the collected products were washed with water and ethanol and dried at 60 °C for 10 h. To produce crystalline SiO_2_@α-Fe_2_O_3_ the samples were calcined at 450 °C for 2 h.

Ag nanoparticles were photo-deposited by dispersing SiO_2_@α-Fe_2_O_3_ nanocomposite spheres in a 50 mL beaker with the addition of 10 mL distilled water and 10 mL ethanol. After 30 minutes of vigorous stirring, 1 wt% of AgNO_3_ were added and stirring extended for another 30 min. The above solution was stirred continuously for 60 min under UV light and the final product was obtained by centrifugation and washed with water and ethanol. The Ag photo-deposited SiO_2_@α-Fe_2_O_3_ solid products were dried in a vacuum oven at 60 °C for 6 h. For comparison of the effect of the loading of Ag on the SiO_2_@α-Fe_2_O_3_ nanocomposites, nanoparticles with different weight percentages of Ag nanoparticles (2 and 3) were also prepared by the above method.

## 3. Characterization

The crystallographic structure of the Ag-SiO_2_@α-Fe_2_O_3_ nanocomposite spheres was analyzed by X-ray diffraction (XRD, with an Analytical X'Pert PRO) using filtered Cu-K_α_ radiation (*λ* = 1.5418 Å) operated at a voltage of 45 kV and current of 40 mA. The morphology of as-prepared samples were studied and structural analysis were carried out using field emission scanning electron microscopy (JEOL JSM-7610) with energy dispersive X-ray analysis (EDX). XRF (X-ray fluorescence S_2_ PICOFOX) is used to determine the chemical composition of Ag-SiO_2_@α-Fe_2_O_3_ nanocomposite spheres. The UV-vis diffuse reflectance spectra were captured by a UV-vis spectrometer (Agilent Cary 5000) using a reference of wavelength of 200–800 nm and Raman Spectroscopy was carried out using DONGWOODM500i, Gyeonggibo, Korea. Electrochemical impedance spectroscopy (EIS) was observed using CHI614c electrochemical analyzer. In this measurement a glassy carbon electrode (GCE) was used as a working electrode and platinum wire and an Ag/AgCl electrode (Sat. KCl) were used as counter and reference electrodes, respectively. The temperature programmed reduction of hydrogen (H_2_-TPD) was measured in H_2_ at the heating rate of 20 °C min^−1^.

### 3.1 Reduction of CO_2_ using *in situ* FTIR

In this study, the *in situ* FTIR spectrometer was fitted with a DRIFT (PerkinElmer FT-IR Spectrometer LX 10-8873, Frontier). The DRIFT cell was overlapped by a 3-window dome with two of the windows allowing for IR transparency and one for UV light radiation. The catalytic activity of the Ag-SiO_2_@α-Fe_2_O_3_ nanocomposite spheres was monitored by analyzing the gas composition during CO oxidation. The DRIFT experiments were carried out from room temperature to 350 °C to find compositional changes on the catalyst surface. The Ag-SiO_2_@α-Fe_2_O_3_ nanocomposite spheres were examined with initial concentration of 2% in air and a flow rate off of 50 standard cubic centimeter per minute (SCCM).

## 4. Results and discussion

### 4.1 X-ray diffraction

**Fig. 1:**
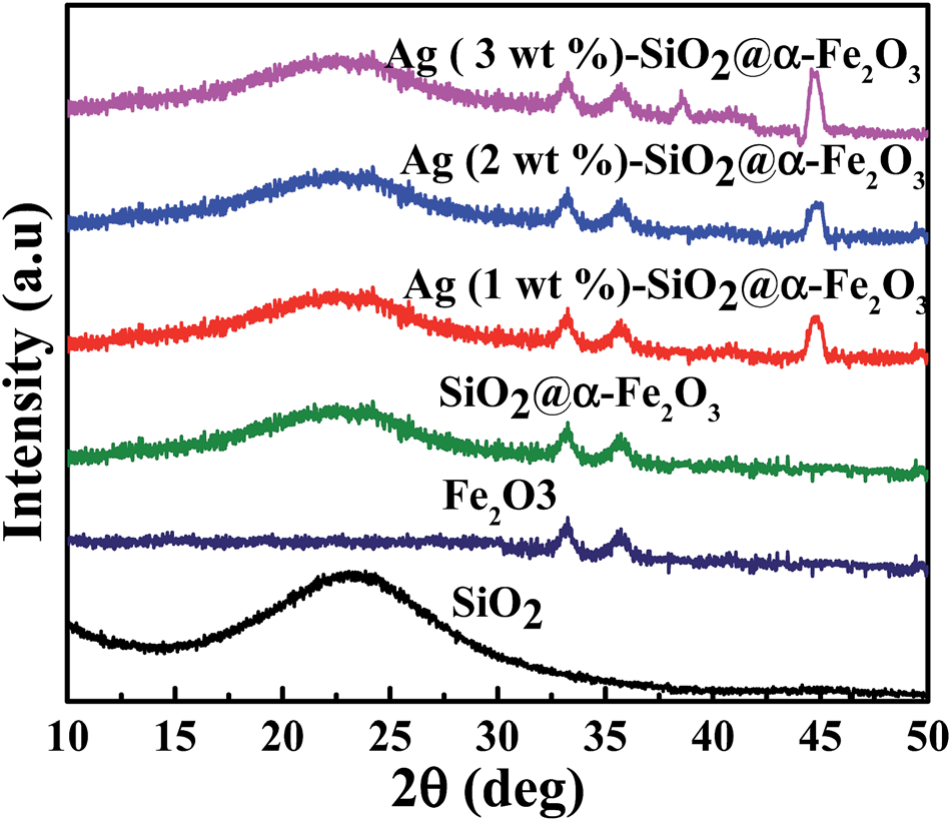
XRD spectra of SiO_2_, α-Fe_2_O_3_, SiO_2_@α-Fe_2_O_3_ and 1, 2 and 3 wt% Ag deposited SiO_2_@α-Fe_2_O_3_ nanocomposites.

The crystal phase structures of SiO_2_, α-Fe_2_O_3_, SiO_2_@α-Fe_2_O_3_ and 1, 2 and 3 wt% Ag deposited SiO_2_@α-Fe_2_O_3_ nanocomposite were investigated by XRD measurement, as shown in Fig. 1. The broad peaks observed at 24° correspond to the SiO_2_ nanocomposite spheres assigned to the partial crystallized material. Two diffraction peaks at 32° and 35.7° indicate the hematite phase (α-Fe_2_O_3_) of the reference profile: JCPDS number 33-0664.^42^ No other extra peaks were observed and the intensity of the SiO_2_ and α-Fe_2_O_3_ ascribed to the standard planes of the SiO_2_ and α-Fe_2_O_3_ showed that maintenance of the structure of the SiO_2_ after the deposition of α-Fe_2_O_3_. The Ag-SiO_2_@α-Fe_2_O_3_ spectrum of the Ag nanoparticles shows the appearance of two diffraction peaks for the nanocomposites at 38.2° and 44.3° which can be ascribed to the (111) and (200) planes. This confirms the formation of the active phase in the Ag nanoparticles on the SiO_2_@α-Fe_2_O_3_ surface.^45^ The weak diffraction peak for the 1 wt% Ag indicates the low loading of Ag on the composites spheres. After increasing the Ag content to 2 and 3 wt%, the intensity became higher as the Ag nanoparticle content increased.

### 4.2 FE-SEM analysis

**Fig. 2:**
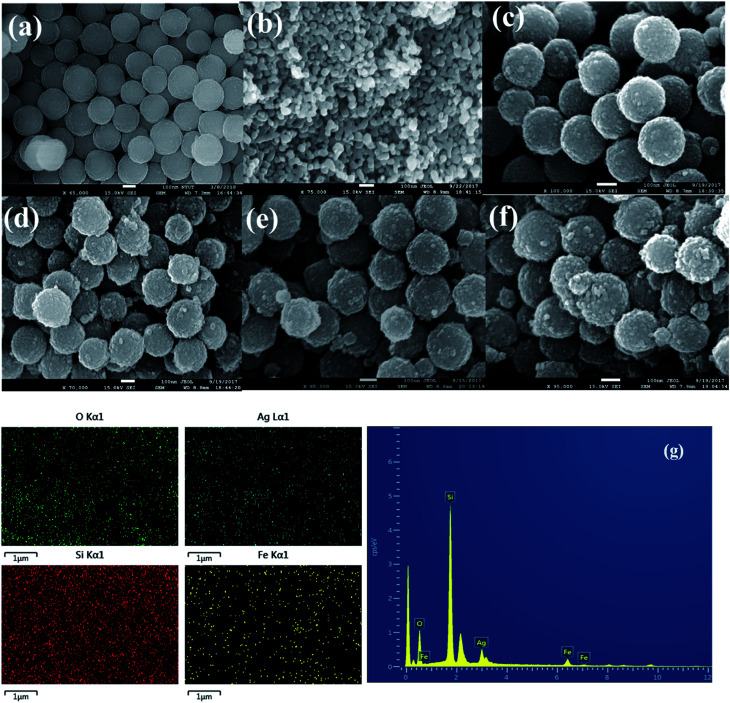
FE-SEM picture of (a) SiO_2_ (b) α-Fe_2_O_3_, (c) SiO_2_@α-Fe_2_O_3_ (d) Ag (1 wt%)-SiO_2_@α-Fe_2_O_3_, (e) Ag (2 wt%)-SiO_2_@α-Fe_2_O_3_, (f) Ag (3 wt%)-SiO_2_@α-Fe_2_O_3_ nanocomposite and (g) EDX spectra of Ag 3 wt %-SiO_2_@α-Fe_2_O_3_ nanocomposite.

FE-SEM analysis was performed to show the surface morphology of the α-Fe_2_O_3_ and different weight percentage of Ag nanoparticles on SiO_2_@α-Fe_2_O_3_ nanocomposites. In Fig. 2(a) and (b) shows the SiO_2_ nanospheres and the α-Fe_2_O_3_ nanoparticles that are nearly uniform in size and highly ordered. Fig. 2b shows the distribution of the α-Fe_2_O_3_ nanoparticles on SiO_2_ sphere which had an average size of 20–25 nm. After deposition of the α-Fe_2_O_3_ nanoparticles over SiO_2_, there is not change in shape of the spheres but the surface did change from smooth to rough, suggesting the formation of a thin layer of α-Fe_2_O_3_ on the surface of the SiO_2_. Fig. 2d–f show the 1, 2 and 3 wt% Ag deposited SiO_2_@α-Fe_2_O_3_ nanocomposite spheres, respectively. Ag-nanoparticle sizes of 10 to 30 nm can be detected. The Ag nanoparticles within the range of 2 to 50 nm in size are highly interactive producing the plasmonic effect.^46^ For 1 and 2 wt% Ag deposited on SiO_2_@α-Fe_2_O_3_, the Ag nanoparticles are homogeneously deposited on the surface of the SiO_2_@α-Fe_2_O_3_ whereas for the 3 wt% Ag nanoparticles they are aggregated into bigger particles almost covering the SiO_2_ and α-Fe_2_O_3_ particles. The energy dispersive X-ray analysis (EDX) results for the Ag-SiO_2_@α-Fe_2_O_3_ nanocomposites are shown in Fig. 2f. The mapping confirms the distribution of the α-Fe_2_O_3_ on the SiO_2_ spheres and Ag nanoparticles dispersed on the surface of the α-Fe_2_O_3_ nanocomposites. XRF analysis was carried out to observe the elemental composition of Ag-SiO_2_@ α-Fe_2_O_3_ nanocomposites. It shows the presence of Si, Fe, and Ag metals confirming the existence of these elements in the nanocomposites. From Table 1 it can be revealed that the 1, 2 and 3 wt% of Ag with respect to α-Fe_2_O_3_ was present and there is a no metals impurities are observed in the nanocomposites.

**Table 1:** XRF analysis of the Ag-SiO_2_@α-Fe_2_O_3_, nanocomposites with different Ag weight percentage

Samples	Si concentration	Fe concentration	Ag concentration
SiO_2_@α-Fe_2_O_3_	93.25	6.753	0
Ag (1 wt%)-SiO_2_@α-Fe_2_O_3_	92.18	6.756	0.071
Ag (2 wt%)-SiO_2_@α-Fe_2_O_3_	92.25	6.751	0.147
Ag (3 wt%)-SiO_2_@α-Fe_2_O_3_	91.65	6.753	0.210

### 4.3 UV-visible spectrophotometer

**Fig. 3:**
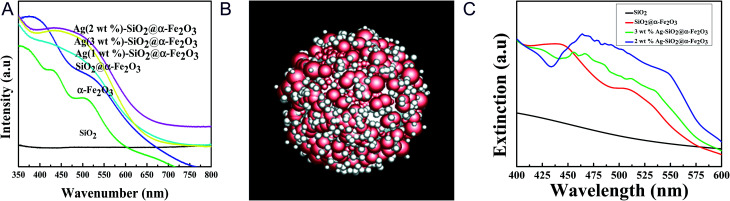
(a) UV-vis spectra of SiO_2_, α-Fe_2_O_3_, SiO_2_@α-Fe_2_O_3_ and 1, 2 and 3 wt% Ag deposited SiO_2_@α-Fe_2_O_3_ nanocomposites. (b) Simulation geometry with Ag nanoparticles. The rough thickness of the red α-Fe_2_O_3_ shell is about 25 nm and the radius of the white SiO_2_ core is 125 nm. (c) UV-vis simulation spectra of SiO_2_, SiO_2_@Fe_2_O_3_ and Ag with radius of 15 nm and 40 nm deposited SiO_2_@Fe_2_O_3_ nanocomposites.

Fig. 3a shows the UV-vis spectroscopic results for the SiO_2_ spheres, α-Fe_2_O_3_, SiO_2_@α-Fe_2_O_3_, and Ag-SiO_2_@α-Fe_2_O_3_ composite spheres with different Ag percentages for investigation of the plasmonic properties as a nanocomposite catalyst. The peaks at 450 and 500 nm are confirming the absorbance of α-Fe_2_O_3_ (ref. 44) and there is no observable peak for SiO_2_ sphere which is due to the insulating material. For the Ag (1 wt%)-SiO_2_@α-Fe_2_O_3_ sample, the absorbance peak at 525 nm can be attributed to the SPR effect of Ag nanoparticles, which is confirmed by D. Philip *et al.*^47,48^ An intense band centered at 525 nm related to metal charge transfer in the SiO_2_@α-Fe_2_O_3_ structure given the existence of Ag nanoparticles. Strong absorption of visible light was observed for the core–shell nanocomposites of Ag-SiO_2_@α-Fe_2_O_3_ due to localized surface plasmon resonance (SPR) with excitation of the electrons from the conduction band to the valance band and suppression of the charge recombination rate. The plasmon resonance results showed the samples with 1 and 2 wt% Ag nanoparticles coated over SiO_2_@α-Fe_2_O_3_ to be more active in the visible region, giving broader absorption. The active plasmonic effect was observed for the lower amounts and smaller sized Ag nanoparticles. For the Ag 3 wt% samples, the sizes of the particles were larger and with greater aggregation with one another, which decrease the SPR effect. Furthermore, the higher loading of Ag caused the accumulation and creation of new recombination centers leading to suspension of electron and charge transfer.

It is generally observed that the transfer of electron density by chemisorbed ligands results in an effect of changes in plasmon band position for the Ag nano particles with the sizes of about 5–50 nm.^18,49,50^ In Fig. 3a, the absorbance at 525 nm observed by the effect of Surface Plasmon Resonance (SPR) for different concentration of Ag nanoparticle on SiO_2_@α-Fe_2_O_3_ nanocomposite with the Ag nanoparticles sizes of 10, 20 and 40 nm. The Ag nanoparticles on the α-Fe_2_O_3_ photocatalyst enhances the visible light absorption and it is useful for increasing the utilization efficiency of solar light.^19–21^ When the concentration of Ag nanoparticles increased from 1 to 2 wt%, the Ag nanoparticles sizes are increased as 10 and 20 nm, respectively. This increase leads to the optical scattering and the electron fields are spatially non-homogeneous. Further, it was found that the possibility of energy transfer between Ag and semiconductor can be maximally enhanced when the size of the metal particles are around 20 nm.^51^ In addition, when plasma induced photoexcited from SRP effect, the electrons transfer to the conduction band of α-Fe_2_O_3_ that enhances the photocatalytic activity when irradiates the visible light. When particles sizes are above 40 nm with accumulation, the 3 wt% Ag nanoparticles are not strong enough to excite the electron to the semiconductor and the electron–hole pair recombination is occurred, thus leads to lower the plasmonic effect.

We stimulated the UV-visible spectra to observe the real conditions of the plasmonic effects ascribed to the different weight percentages of Ag nanoparticles as shown in Fig. 3b. In order to understand the effect of the silver nanoparticles on the extinction spectra of the Ag-SiO_2_@α-Fe_2_O_3_-shell nanoparticle, finite-difference time-domain (FDTD) method is adopted which solves the Maxwell's equations. Fig. 3b is the core shell nanoparticle without Ag, where the thickness of α-Fe_2_O_3_ rough shell (red spheres) is around 40 nm and the SiO_2_ core (white core) has a radius of 125 nm. Fig. 3b is the core shell nanoparticle with Ag, where the radii of the random silver nanoparticles are 10 nm and 15 nm. The simulation domain is 1.5 μm × 1.5 μm × 1.5 μm with uniform mesh, Δ*x* = Δ*y* = Δ*z* = 3 nm. The refractive index of α-Fe_2_O_3_ is set according to M. Gartner *et al.*^52^ The refractive indexes of SiO_2_ and Silver are fitted by a FDTD package Lumerical Inc. according to Palik and Johnson and Christy's experiment results. The total field scattered field is selected and the wavelength is set from 300 nm to 800 nm. There are six monitors inside the source to collect the absorption power and six monitors outside the source to collect the scattering power. The extinction power is the sum of the absorption power and the scattering power. Fig. 3c shows the extinction power of SiO_2_, SiO_2_@α-Fe_2_O_3_, Ag-SiO_2_@α-Fe_2_O_3_ with *r*_ag_ = 20 nm, and Ag-SiO_2_@α-Fe_2_O_3_ with *r*_ag_ = 7 nm. Adding silver nanoparticles reduces the scattering power in short wavelength around 300 nm but increases the absorption power in a broad range from 400 nm to 600 nm. Comparing to the UV-vis Spectra in Fig. 3a, the plasmonic effect is confirmed with a similar trend.

### 4.4 Raman Spectroscopy

**Fig. 4:**
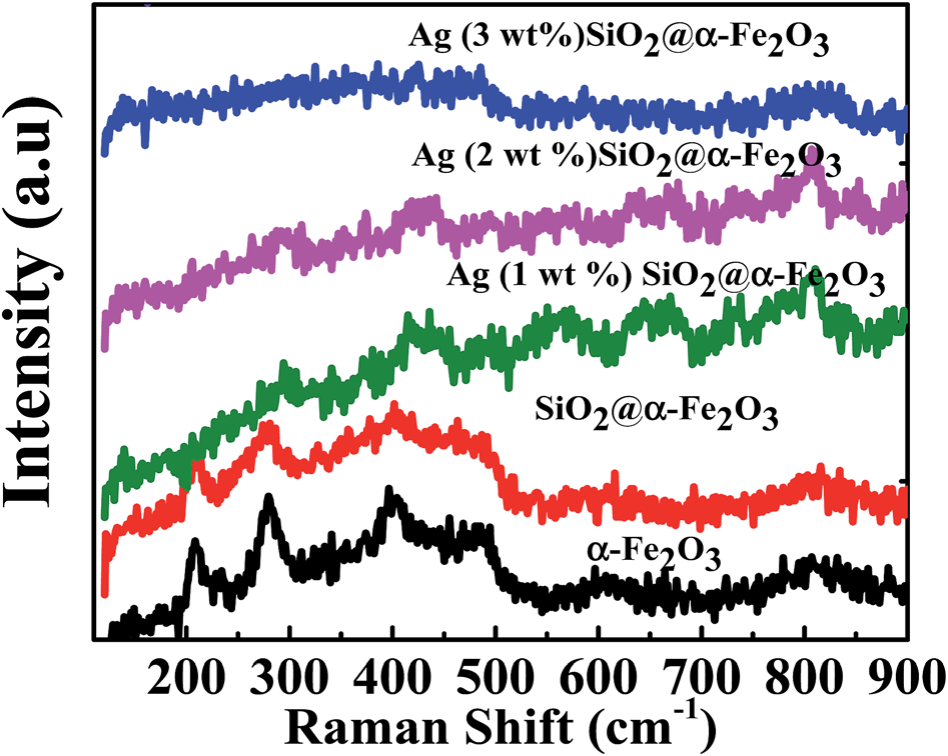
Raman spectra of the SiO_2_, α-Fe_2_O_3_, SiO_2_@α-Fe_2_O_3_, Ag-SiO_2_@α-Fe_2_O_3_ nanocomposite with different Ag weight percentages.

The Raman spectra of the SiO_2_, α-Fe_2_O_3_, SiO_2_@α-Fe_2_O_3_, Ag-SiO_2_@α-Fe_2_O_3_ nanocomposite with different Ag weight percentages are shown in Fig. 4. The strong peaks observed around 225, 247, 299, 412, 497 and 613 cm^−1^ confirm the presence of α-Fe_2_O_3_. The figure shows the clear Raman effects of α-Fe_2_O_3_. The peaks at 305, 513 and 660 cm^−1^ belonging to pure Fe_2_O_3_ were not observed.^53^ However, the Ag-related signal was not observed for 1 and 2 wt% Ag because of the weak Raman scattering except for the 3 wt% Ag nanoparticles. The Raman peak indicates that the presence of α-Fe_2_O_3_ on the surface of the α-Fe_2_O_3_ without any phase change.

### 4.5 FTIR spectroscopy results

**Fig. 5:**
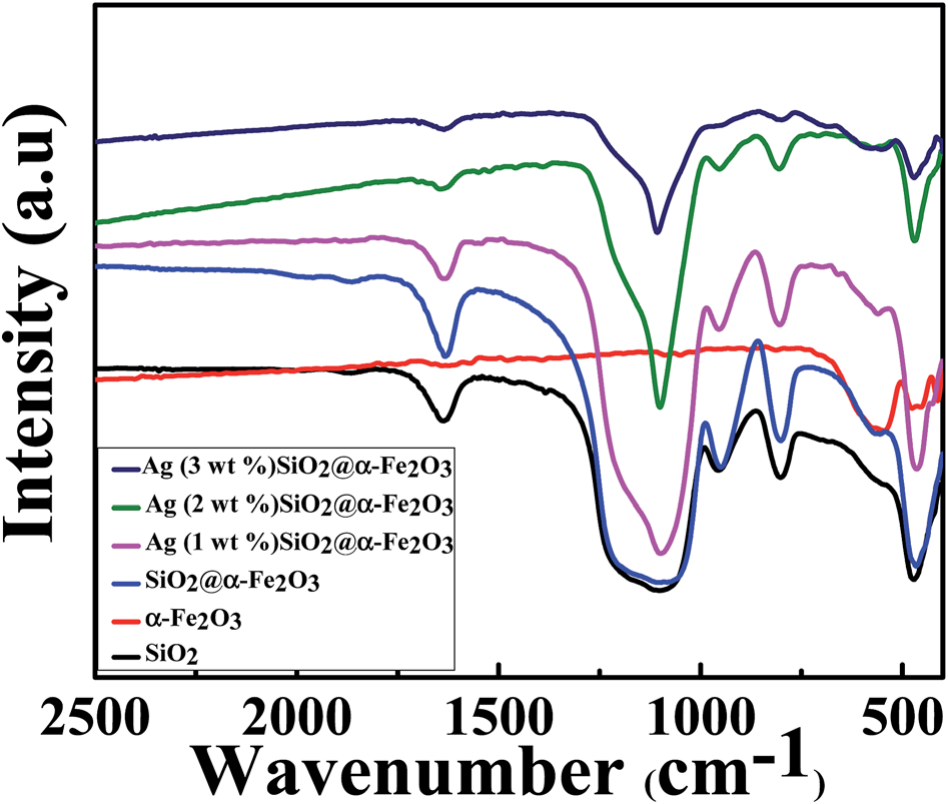
FT-IR spectra of the SiO_2_, α-Fe_2_O_3_, SiO_2_@α-Fe_2_O_3_, Ag-SiO_2_@α-Fe_2_O_3_ nanocomposites with different Ag weight percentages.

Fig. 5 shows the chemical structure of the SiO_2_, α-Fe_2_O_3_, SiO_2_@α-Fe_2_O_3_, Ag-SiO_2_@α-Fe_2_O_3_ nanocomposites with different Ag weight percentages. The peak at 1080–1105 cm^−1^ demonstrates the symmetric vibration of Si–O–Si and 940–960 cm^−1^ which can be assigned to the Si–OH vibration. The absorption band at 558 cm^−1^ in the curve is attributed to the bending vibrations of the Fe–O in α-Fe_2_O_3_ spectra. In addition, the absorption band at about 685 cm^−1^, observed in α-Fe_2_O_3_ that can be assigned to the bending modes of Fe–O–H.^54^ Moreover, there is not much difference in chemical structure of the SiO_2_@α-Fe_2_O_3_ composites spheres with various Ag compositions and indicates that the different weight percentages of Ag did not interfere with the structure of the SiO_2_@α-Fe_2_O_3_. This means that the catalytic active force between Ag and SiO_2_@α-Fe_2_O_3_ nanocomposites is a physical process, rather than chemical reaction.

### 4.6 PL spectroscopy

**Fig. 6:**
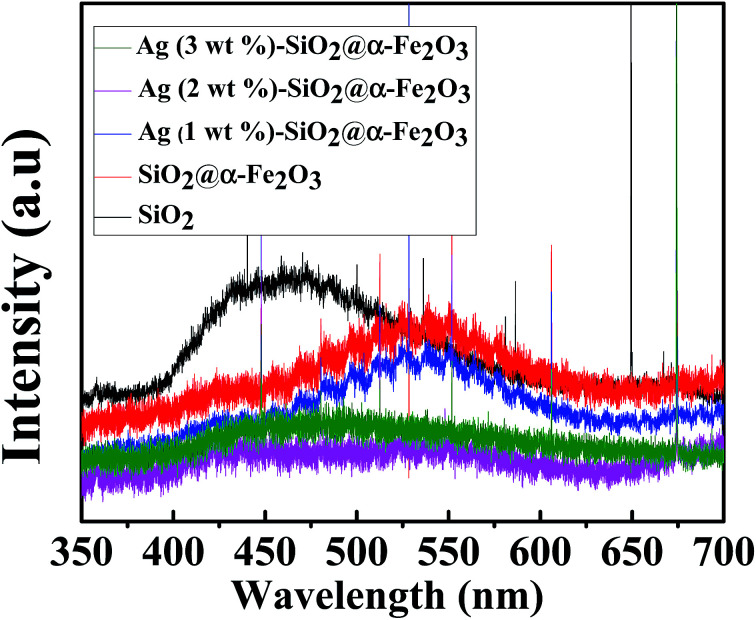
PL spectra for SiO_2_ sphere, α-Fe_2_O_3_, SiO_2_@α-Fe_2_O_3_, SiO_2_@α-Fe_2_O_3_, Ag (1 wt%)-SiO_2_@α-Fe_2_O_3_, Ag (2 wt%)-SiO_2_@α-Fe_2_O_3_ and Ag (3 wt%)-SiO_2_@α-Fe_2_O_3_ samples.

The PL spectra of SiO_2_ sphere, SiO_2_@α-Fe_2_O_3_, SiO_2_@α-Fe_2_O_3_, Ag (1 wt%)-SiO_2_@α-Fe_2_O_3_, Ag (2 wt%)-SiO_2_@α-Fe_2_O_3_ and Ag (3 wt%)-SiO_2_@α-Fe_2_O_3_ samples are shown in Fig. 6. The SiO_2_ sphere shows the broad-band emission around 450 nm. In SiO_2_@α-Fe_2_O_3_ nanocomposites, the emission at 540 nm can be assigned to the recombination of photoexcited holes with electrons inhabiting the singly ionized oxygen vacancies in α-Fe_2_O_3_.^55^ After depositing Ag nanoparticles, the recombination peak at 540 nm is largely decreased, indicating that the recombination of electron–hole pairs has been reduced. Ag (2 wt%) SiO_2_@α-Fe_2_O_3_ nanocomposites shows minimum intense PL spectra. This low intensity suggests that the surface modified Ag (3 wt%) SiO_2_@α-F_e2_O_3_ nanocomposites have significant recombination of photogenerated electrons–hole pairs when compared with other two Ag percentages.

### 4.7 Electrochemical impedance study

**Fig. 7:**
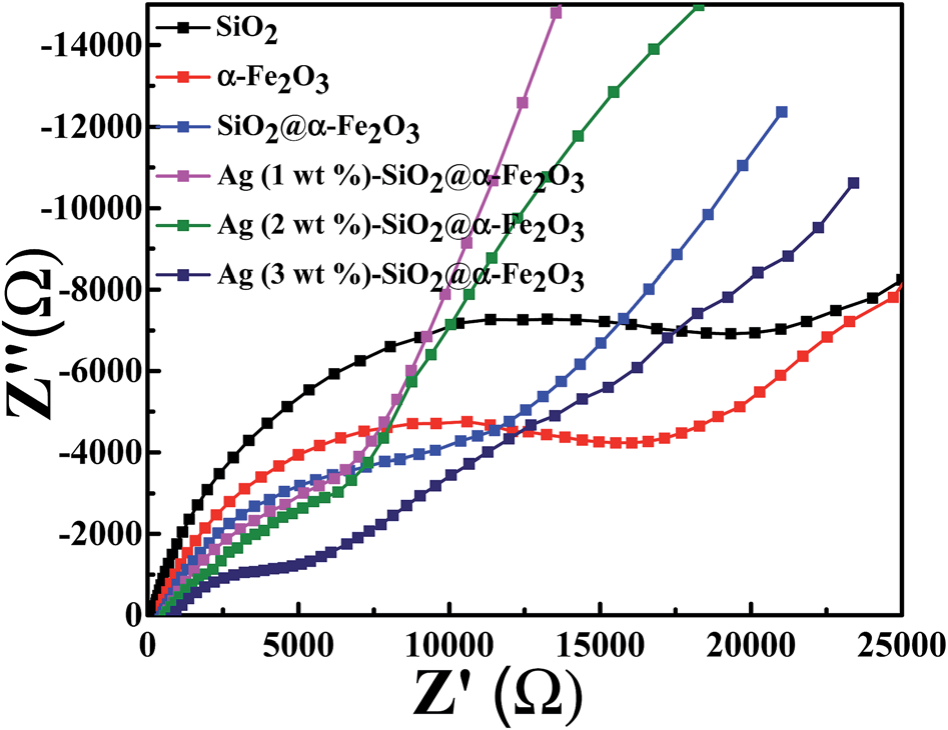
Electrochemical impedance spectra for SiO_2_ sphere, α-Fe_2_O_3_, SiO_2_@α-Fe_2_O_3_, SiO_2_@α-Fe_2_O_3_, Ag (1 wt%)-SiO_2_@α-Fe_2_O_3_, Ag (2 wt%)-SiO_2_@α-Fe_2_O_3_ and Ag (3 wt%)-SiO_2_@α-Fe_2_O_3_ samples.

Electrochemical impedance measurement was also performed for the SiO_2_ sphere, α-Fe_2_O_3_, SiO_2_@α-Fe_2_O_3_, SiO_2_@α-Fe_2_O_3_, Ag (1 wt%)-SiO_2_@α-Fe_2_O_3_, Ag (2 wt%)-SiO_2_@α-Fe_2_O_3_ and Ag (3 wt%)-SiO_2_@α-Fe_2_O_3_ samples as shown in Fig. 7. The small semicircles observed for the 2 wt% of Ag showing the electron transfer impedance of the electrode surface specifying the better charge transfer resistance on the Ag (2 wt%)-SiO_2_@α-Fe_2_O_3_ interface. The diameter of the arc radius on the EIS Nyquist plot is very low due to a more effective separation of photogenerated electron–hole pairs and the faster interface charge transfer rate which appeared for the 2 wt% Ag nanoparticles SiO_2_@α-Fe_2_O_3_ composites spheres. It is also worth mentioning that the higher impedance values produced for the 2 wt% Ag on SiO_2_@α-Fe_2_O_3_ composites spheres when compared to 1 and 3 wt% Ag on SiO_2_@α-Fe_2_O_3_ composites spheres. The improvement in the impedance of the charge transfer of electrons from the SiO_2_@α-Fe_2_O_3_ to Ag nanoparticles is confirmed by the UV-vis spectra as shown in Fig. 3

### 4.8 Temperature programmed desorption (H_2_-TPD)

**Fig. 8:**
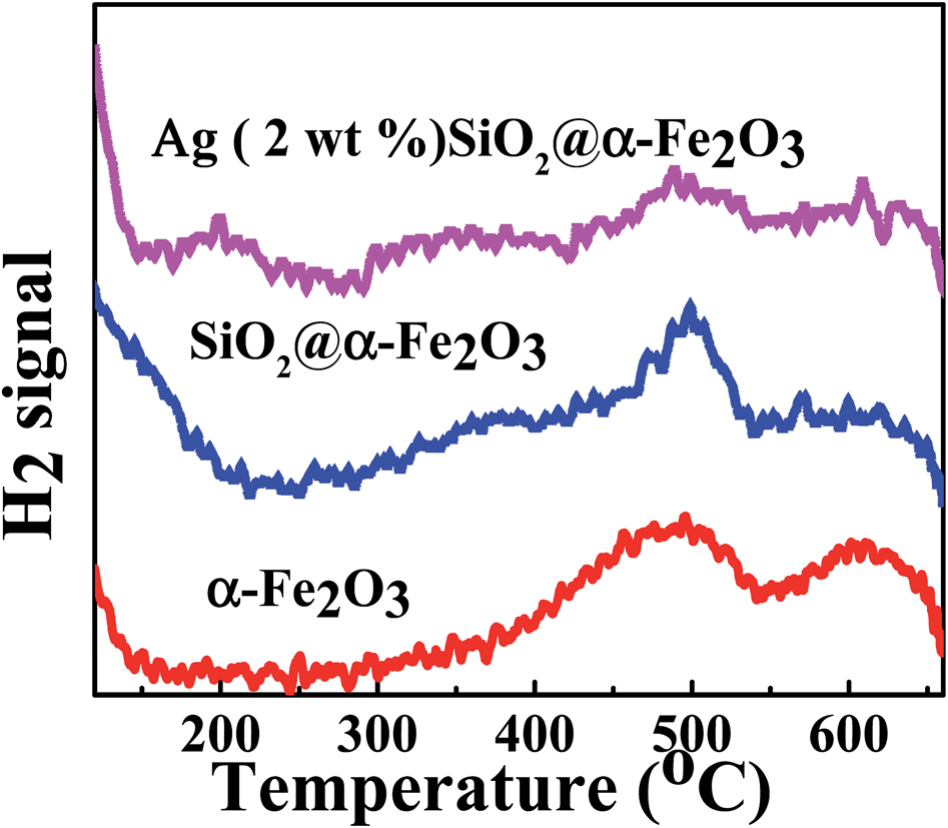
H_2_-TPD profiles of the α-Fe_2_O_3_, SiO_2_@α-Fe_2_O_3_ and Ag (2 wt%)-SiO_2_@α-Fe_2_O_3_ nanocomposite spheres.

Fig. 8 shows the H_2_-TPD profiles of the α-Fe_2_O_3_, SiO_2_@α-Fe_2_O_3_ and Ag (2 wt%)-SiO_2_@α-Fe_2_O_3_ nanocomposite spheres. For α-Fe_2_O_3_ and SiO_2_@α-Fe_2_O_3_, we can realize that the peak maximized at 500 °C is assigned to the reduction of Fe_2_O_3_ to Fe_3_O_4_ as confirmed in previous work.^56^ The other peak at 600 °C attributed to FeO to Fe^0^. In addition, the weak peak observed around 200 °C is probably arises from the movement of Ag nanoparticles to the adjacent Ag-SiO_2_@α-Fe_2_O_3_ nanocomposites. It indicates that after adding Ag nanoparticles, the catalytic activity increases that leads to the CO oxidation due to high plasmonic effect.

### 4.9 Photocatalytic oxidation of CO examined with DRIFT

**Fig. 9:**
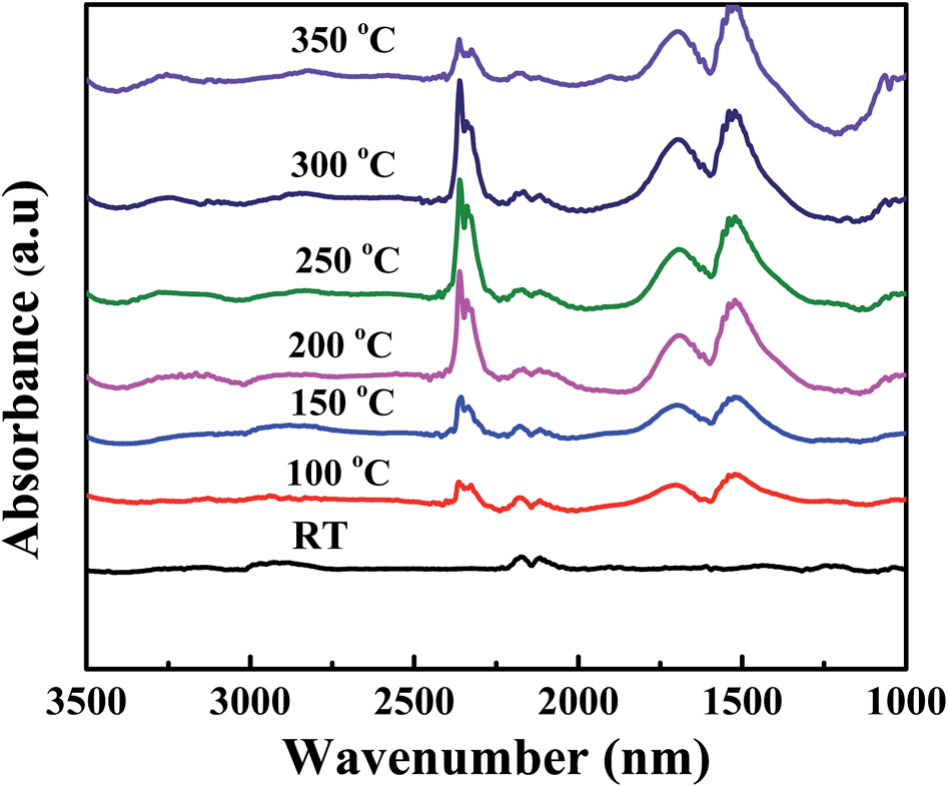
DRIFT spectra of Ag (2 wt%) SiO_2_@α-Fe_2_O_3_ nanocomposites measured at different temperature during CO oxidation.

The DRIFT was monitored to collect information about the temperature dependent gaseous phase on Ag (2 wt%) SiO_2_@α-Fe_2_O_3_ nanocomposites during CO oxidation reaction as shown in Fig. 9. With the introduction of CO to the Ag (2 wt%)-SiO_2_@α-Fe_2_O_3_ nanocomposites at room temperature, peaks appear at 2176 and 2142 cm^−1^ which are due to the gaseous carbon monoxide doublet. In Fig. 9, as the temperature increases to 100 °C, new peaks appear at 2353 cm^−1^ and 2362 cm^−1^_,_ implying CO_2_ absorption and adsorbed doublets of carbon dioxide which are visible in the spectrum. The CO_2_ peak reflects the reaction between CO and adsorbed oxygen, which leads to the formation of reactive formats which can be easily oxidized by oxygen absorbed by the silver nanoparticles.^57^ The intensity of carbon dioxide doublet peaks increases with increasing the temperature to 300 °C. Furthermore, new bands also appear at 1621, 1414 and 1222 cm^−1^ indicating the formation of a bicarbonate after the adsorption of CO_2_ on α-Fe_2_O_3_. This confirms that the main reason for the oxidation process is based on the presence of Ag nanoparticles with high CO oxidation activity. These results agree well with the H_2_-TPD results. For the Ag-SiO_2_@α-Fe_2_O_3_ nanocomposite spheres, a high intensity efficient peak at 2353 cm^−1^ and 2362 cm^−1^can be observed at 250 and 300 °C, as shown in the Fig. 8. The bicarbonates remained stable at temperatures up to 350 °C, after which the band at 2134 cm^−1^ was decreased, which can be attributed to the low reactivity between the CO and the adsorbed oxygen. Furthermore, when the temperature was increased to 350 °C there was a very less effective reaction occurring between the CO and adsorbed oxygen due to the lack of the –OH group.

One should note that CO oxidation is based on the exothermic reaction and it generates more –OH groups on the surface of the adsorbent. When CO molecules act as an electron donor that adsorbed on the surface of Ag and oxygen in the Ag are responsible for the oxidation of CO. During the photo oxidation of CO using different concentration of Ag on SiO_2_@α-Fe_2_O_3_ nanocomposites catalyst, the SiO_2_ spheres act as a substrate as well as a good absorbent, while the α-Fe_2_O_3_ acts as a semiconductor for the generation of electrons and holes. Moreover, the Ag nanoparticles on the SiO_2_@α-Fe_2_O_3_ catalyst assist the trapping of electrons through the conduction band of α-Fe_2_O_3_ which diminishes the electron–hole recombination rate. However, the presence of Ag nanoparticles on the surface of α-Fe_2_O_3_ generally increases the contact area between Ag nanoparticles and α-Fe_2_O_3_. Further, an Ag atom and CO molecules give a strong interactions between the bridging oxygen and the surface of α-Fe_2_O_3_ due to the plasmonic effect, thus leads to photo oxidation. When the temperature increased from 100 to 350 °C the catalytic activity of Ag nanoparticles generating more and more superoxide radicals which leads to CO oxidation. Interestingly, when temperature increases from 250 to 300 °C the more amount of CO oxidation occurred and high SPR effect from Ag nanoparticles also helped to improve the CO oxidation activity. The plasmonic electron injection from SPR from Ag nano particles leads to generate more resonant photons for photon harvesting, thereby facilitating the photocatalytic process.^47,58^ This work confirmed that the SPR and thermal energy are responsible for the transfer of energetic electrons from the Ag metal to the semiconductor or a consequence of the direct interaction of surface plasmon with adsorbents.^59,60^ The SPR can utilize thermal energy and a low-intensity photon flux to improve the rate of catalytic oxidation reactions at significant temperatures on the surface of the catalysts.

**Fig. 10:**
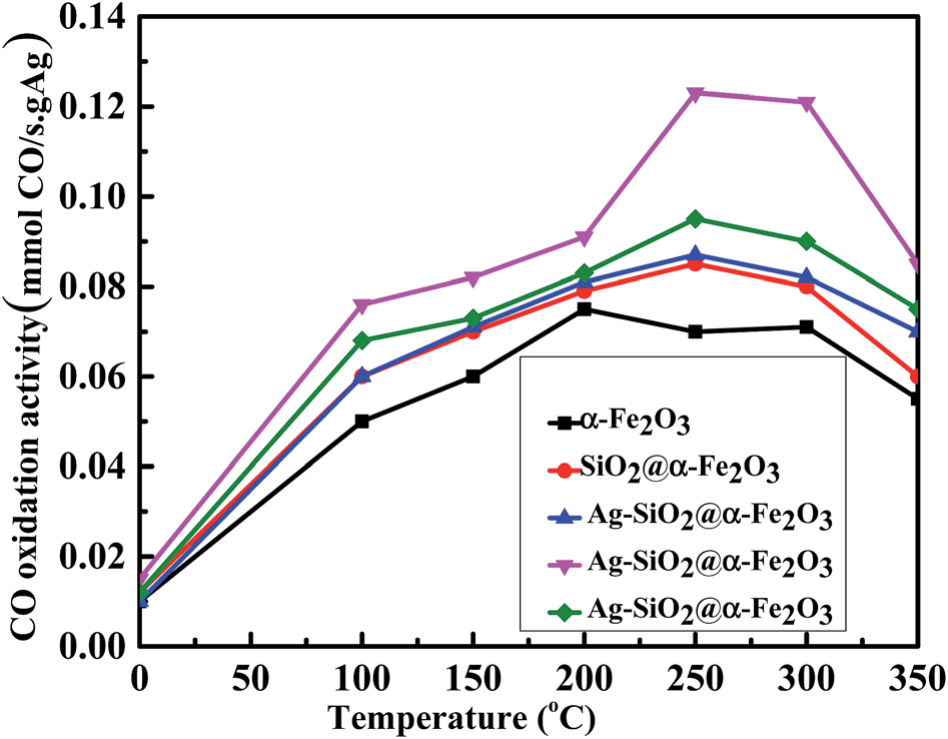
Effect of CO oxidation activity in α-Fe_2_O_3_, SiO_2_@α-Fe_2_O_3_, SiO_2_@α-Fe_2_O_3_, Ag (1 wt%)-SiO_2_@α-Fe_2_O_3_, Ag (2 wt%)-SiO_2_@α-Fe_2_O_3_ and Ag (3 wt%)-SiO_2_@α-Fe_2_O_3_ samples.

Fig. 10 shows the rate of conversion of CO to CO_2_ using 1, 2 and 3 wt% Ag nanoparticles on SiO_2_@α-Fe_2_O_3_ catalysts from room temperature to 350 °C. It is clear that 2 wt% Ag-SiO_2_@ α-Fe_2_O_3_ catalyst activity is more than 48% higher than that of pure Fe_2_O_3_ and SiO_2_@ α-Fe_2_O_3_. The rate of charge-carrier transfer increases during the intermediate plasmon phase between Ag (2 wt%) and the SiO_2_@α-Fe_2_O_3_ nanocomposites. This proves that the CO oxidation readily takes place on the 2 wt% Ag-SiO_2_@α-Fe_2_O_3_ catalyst surface when oxygen is available in atomic form.^61,62^ The CO oxidation rate of 3 wt% Ag is lower when compared to others that are higher recombination rates due to the aggregation of particles with recession in terms of the photo absorption and resonant energy transfer. Further, the photo excitations are dependent on the Ag concentration which reduces the oxidation performance in 3 wt% Ag-SiO_2_@ α-Fe_2_O_3_. This indicates that there is low inter-reaction between the CO and adsorbed oxygen and excess Ag may cover the nanocomposites and hinder direct contact with the SiO_2_@α-Fe_2_O_3_ catalyst.

## 5. Conclusions

A facile method for the photo deposition of different weight percentage Ag nanoparticles on SiO_2_@α-Fe_2_O_3_ nanocomposites was investigated for proper CO oxidation at different temperatures. The most effective CO oxidation was observed to occur at 250 °C with Ag nanoparticles at a weight percentage of 2. The improved photocatalytic activity is attributed to the plasmonic effect of the Ag and the charge transfer property. The computation method proved that the high plasmonic effect was observed on the Ag (2 wt%)-SiO_2_@α-Fe_2_O_3_ nano composites materials. The Ag loaded SiO_2_@α-Fe_2_O_3_ catalyst is shown to be a promising material for conversion of solar energy into CO oxidation.

## Conflicts of interest

There are no conflicts to declare.

## Supplementary Material
